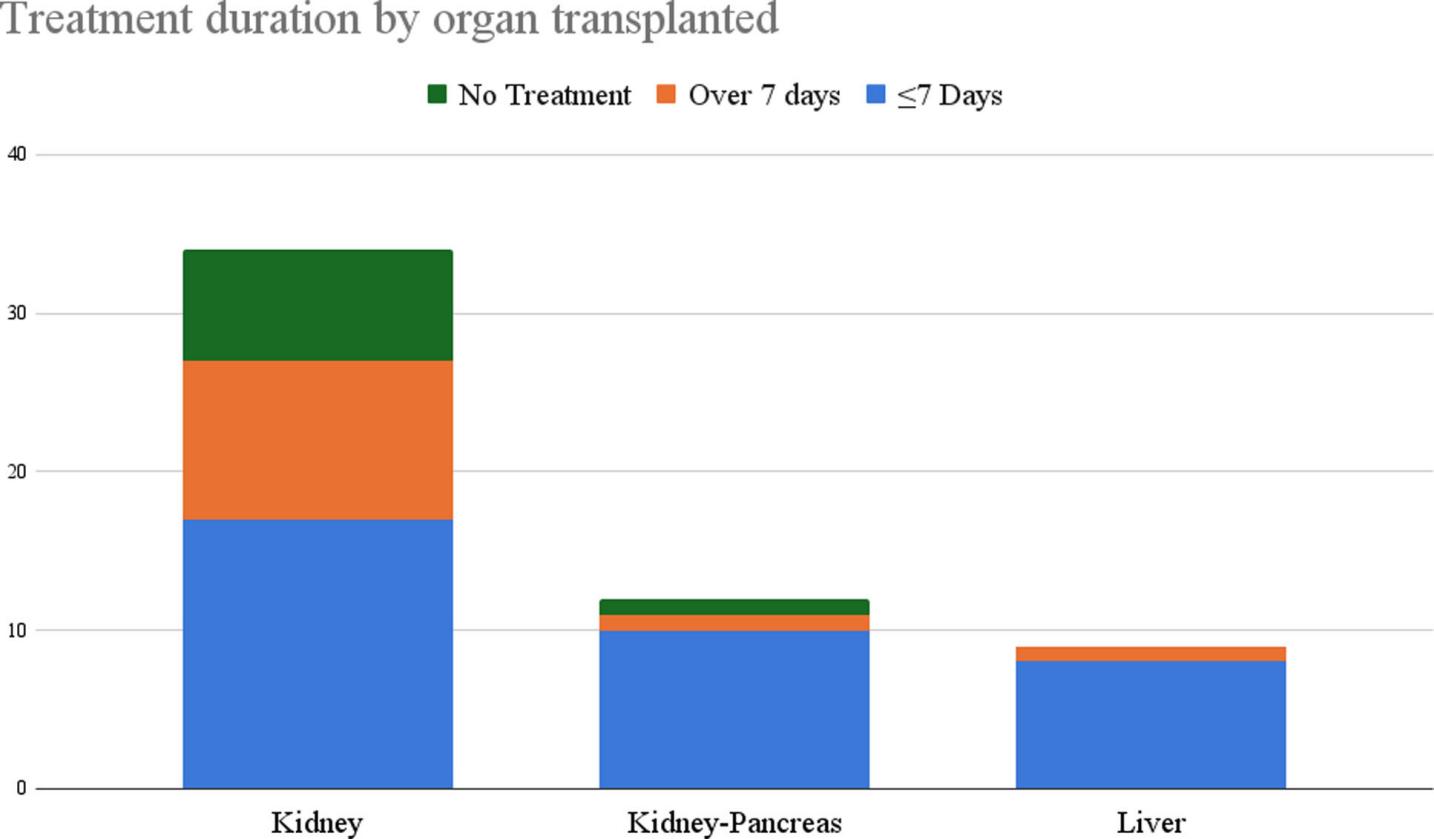# 275 Effect of a Diagnostic Order Set on Sexually-Transmitted Infection Co-Testing

**DOI:** 10.1017/ash.2026.10637

**Published:** 2026-06-23

**Authors:** Lior Cohen Yatziv, Scott Borgetti, Alfredo Mena Lora, Christine Zaky, Gabriela Mendez Gonzalez

**Affiliations:** 1 University of Illinois Chicago; 2 University of Illinios; 3 University of Illinois at Chicago

## Abstract

**Background:** Candida growth in organ preservation fluid (OPF) is uncommon, with reported incidence ranging up to 4% of OPF. Its clinical significance is unclear, as many recipients remain asymptomatic, while on rare occasions, transmission can lead to severe donor-derived infections, most notably mycotic aneurysms, graft loss, and fatal hemorrhage. The current American Society of Transplantation (AST) guidance recommends prompt antifungal therapy when Candida is identified; however, real-world practice patterns and outcomes are not well described. **Methods:** We conducted a retrospective cohort study of adult solid organ transplant recipients at a tertiary academic center from January 2020 through October 2025. Patients with Candida-positive OPF cultures were identified through the medical record. We collected recipients' and donors' demographics, microbiological profiles, antifungal management, and clinical outcomes. Invasive candidiasis was defined as Candida isolated from blood or deep tissue/fluid within 90 days of transplant. **Results:** We identified 55 solid organ transplant recipients with Candida-positive OPF cultures (34 Kidney, 9 Liver, 12 Kidney-Pancreas). The most common isolates (37 patients) were C. albicans and C. glabrata. Treatment patterns and outcomes varied by organ type. Among Kidney recipients, 20.5% (7/34) received no antifungal therapy; notably, none of these untreated patients developed invasive candidiasis, graft loss, or vascular complications. All Liver recipients (n=9) received antifungal therapy, predominantly for <7 days (n=8). Despite treatment, Liver recipients had high morbidity: 22% (2/9) progressed to invasive candidiasis (peritonitis and vascular arteritis), and 67% (6/9) demonstrated vascular Doppler abnormalities. Median length of hospital stay was similar across treatment durations (p < 0.05) and similarly no increased readmission rate among all transplant groups. **Conclusion:** Our findings suggest that the risk of Candida-positive OPF is not uniform across organ types, indicating that current universal treatment recommendations may lead to over-treatment in some populations. Kidney recipients demonstrated favorable outcomes even without antifungal therapy. In contrast, liver recipients remained at risk for significant vascular morbidity and invasive infection regardless of treatment. Shifting to an organ-specific, risk-stratified management strategy can improve antifungal stewardship while ensuring safety for higher-risk recipients.

**Figure:**
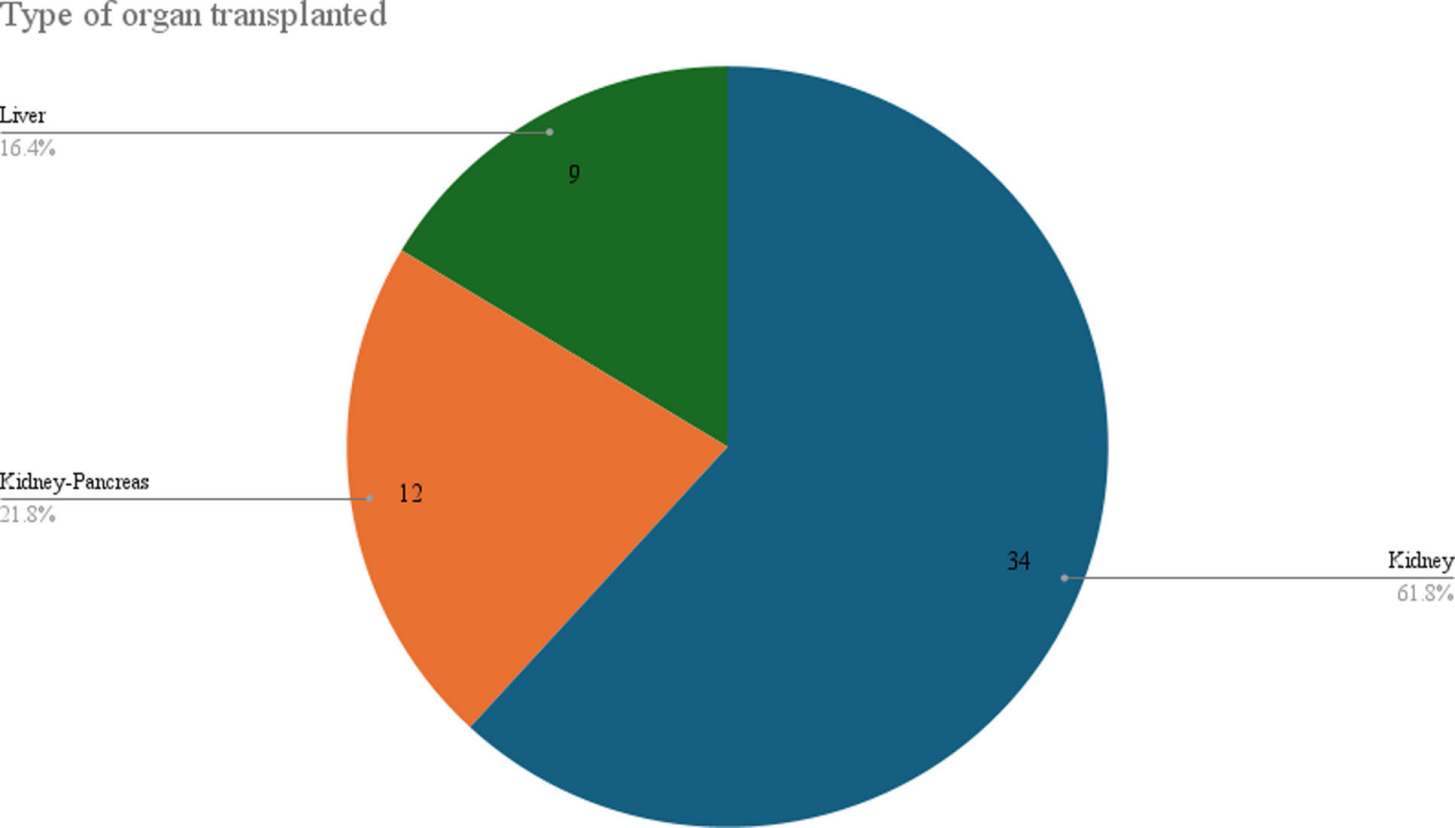

**Figure:**
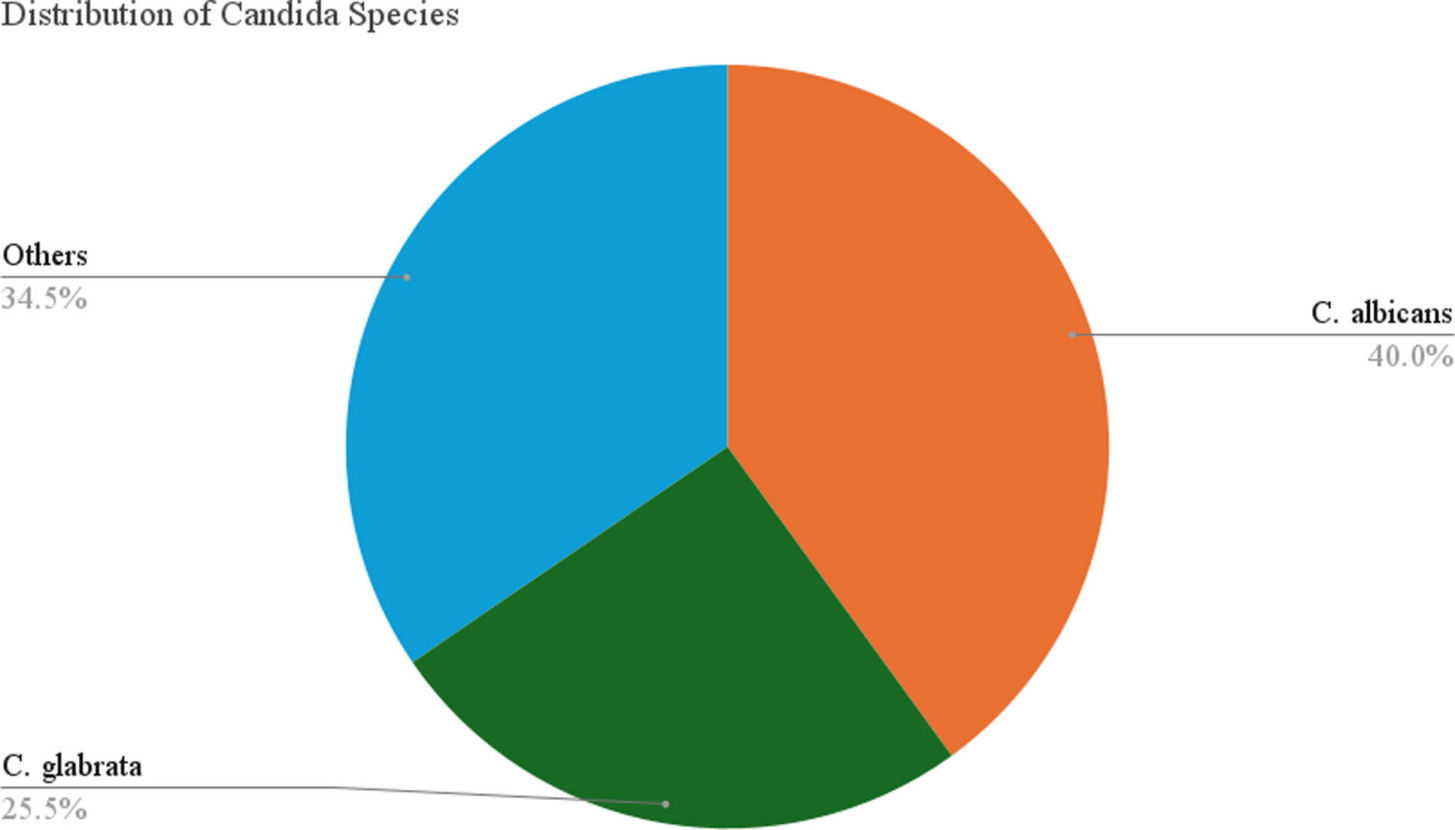

**Figure:**